# A Portable Array-Type Optical Fiber Sensing Instrument for Real-Time Gas Detection

**DOI:** 10.3390/s16122087

**Published:** 2016-12-08

**Authors:** San-Shan Hung, Hsing-Cheng Chang, I-Nan Chang

**Affiliations:** 1 Department of Automatic Control Engineering, Feng Chia University, Taichung 40724, Taiwan; hcchang@fcu.edu.tw; 2 Facilities Management Center, Feng Chia University, Taichung 40724, Taiwan; enchung@fcu.edu.tw

**Keywords:** optical fiber array, Bragg grating, long-period fiber sensing, optical polishing, fluorescence reaction, gases measurement

## Abstract

A novel optical fiber array-type of sensing instrument with temperature compensation for real-time detection was developed to measure oxygen, carbon dioxide, and ammonia simultaneously. The proposed instrument is multi-sensing array integrated with real-time measurement module for portable applications. The sensing optical fibers were etched and polished before coating to increase sensitivities. The ammonia and temperature sensors were each composed of a dye-coated single-mode fiber with constructing a fiber Bragg grating and a long-period filter grating for detecting light intensity. Both carbon dioxide and oxygen sensing structures use multimode fibers where 1-hydroxy-3,6,8-pyrene trisulfonic acid trisodium salt is coated for carbon dioxide sensing and Tris(2,2′-bipyridyl) dichlororuthenium(II) hexahydrate and Tris(bipyridine)ruthenium(II) chloride are coated for oxygen sensing. Gas-induced fluorescent light intensity variation was applied to detect gas concentration. The portable gas sensing array was set up by integrating with photo-electronic measurement modules and a human-machine interface to detect gases in real time. The measured data have been processed using piecewise-linear method. The sensitivity of the oxygen sensor were 1.54%/V and 9.62%/V for concentrations less than 1.5% and for concentrations between 1.5% and 6%, respectively. The sensitivity of the carbon dioxide sensor were 8.33%/V and 9.62%/V for concentrations less than 2% and for concentrations between 2% and 5%, respectively. For the ammonia sensor, the sensitivity was 27.78%/V, while ammonia concentration was less than 2%.

## 1. Introduction

In recent years, flourishing industrial technology development incurs undesirable environmental problems, including severe air pollution, acid rain, global warming, and El Niño. Clean living quality has received significant attention, markedly increasing the needs for gas sensors to monitor environmental quality. Oxygen (O_2_), carbon dioxide (CO_2_), and ammonia (NH_3_) are common indicators of air quality. Consequently, sensors for detecting these gases have been rapidly developed.

Sensors for detecting O_2_, CO_2_, and NH_3_ had been designed separately to measure concerned single gas concentration [1,2,3,4,5,6,7,8,9,10,11,12]. Some harmful gases exit in our living environment to danger our safety. To effectively determine ambient toxic gas conditions and concentrations for decreasing accidents and deaths, developing multifunctional sensing devices to detect different gases simultaneously is of great urgency. Optical fibers are free of electromagnetic interference, small in volume, and easily transferrable. Additionally, such fibers exhibit a wide broadband; hence, they are most applicable to developing sensors. The highly reflective fiber Bragg gratings (FBGs) and phase mask method have been designed, initiating applied research regarding mass-producing fiber grating technology [13,14]. Fiber grating sensors have been prevalently developed and provide various functions such as measuring pressure, sound wave, electrical flow, liquid level, inclined angle, curvature and temperature. Additionally, several fiber grating sensors are designed to bypass environmental interference [15,16,17,18,19,20,21,22,23].

The nano thin film of sol is usually synthesized using tetraethyl orthosilicate (TEOS) and water as the reactants and ammonia or hydrochloric acid as the catalyst. The parameters for synthesizing this type of nano sol-gel have been concluded as concentration, temperature, and pH value, which affect the size and formation time of nano silica particles [24,25,26]. An optical fiber sensor can increase sensitivity by coating a porous nano-sol film. Moreover, applying fluorescent materials to optical fiber sensors can alleviate the problem of light energy loss in evanescent field technology [27,28,29,30,31]. The sensing sol-gel materials are used to provide microporous immobilization support matric to enhance the fluorescence reaction, and also described the approaches to fluorescence-based CO_2_ sensing [32]. There are three operation technologies including fluorescence intensity detection, fluorescence resonance energy transfer (FRET), dual luminophore referencing (DLR), that have been applied to develop optical-fiber CO_2_ sensors [4,5,32]. To obtain responses at a specific frequency, an optical fiber sensor must be connected with a filter for processing the proper signal. Optical fiber filters have been designed based on the optical waveguide technology including the directional coupler and ring resonators [33,34]. A band-pass filter had been developed by coupling double long-period fiber gratings (LPG) with a core mode blocker or a hollow fiber [35,36,37]. Different optical fiber sensing devices for detecting different variables have been developed, such as displacement sensors, strain sensors, pressure sensors and volatile organic compound gas sensors [38,39,40,41,42,43].

In this work, a packaged novel optical fiber sensing array is integrated with signal processing and remote monitoring modules for detecting O_2_, CO_2_, and NH_3_ concentrations in real-time which could possibly be applied in incinerators and dump pits.

## 2. Principle of Fiber Sensing

There are two types of sensors that are integrated design as the proposed optical fiber sensing array. The first type of sensor was installed by integrating FBG and LPG structures to measure NH_3_ gas concentrations by demodulating variations in light intensity with temperature compensation. The sensing region of the NH_3_ sensor was the FBG portion coated with a sensitive nano sol film. By contrast, the O_2_ and CO_2_ sensors were coated with fluorescent nano sol thin films to detect gases through a fluorescence quenching effect [2,4,32].

### 2.1. Principle of Bragg Grating Sensing

The wavelength of the FBG reflection center (λ_B_) is equal to double the product of the equivalent refractive index of fiber core (n_eff_) and the fiber grating period (Λ), as shown in Equation (1):

(1) $$\lambda_{B}=2n_{\mathrm{eff}}\Lambda$$

The fraction of FBG wavelength shift expressed in Equation (2) is induced by the variations of strain and temperature.

(2) $$\frac{\Delta \lambda_{B}}{\lambda_{B}}=(1-P_{e})\Delta \varepsilon +(\alpha_{f}+\xi_{f})\Delta T=K_{\varepsilon }\varepsilon +K_{T}T$$

where Δλ_B_ is the drift of the reflection center wavelength, λ_B_ is the reflection center wavelength, P_e_ is the photoelastic constant (≈0.22), Δε is the stress, α_f_ is the fiber thermal expansion coefficient, ξ_f_ is the fiber thermal optical coefficient (≈6.8 × 10^−6^ °C^−1^) [12], ΔT is the temperature variation, K_ε_ is the stress-sensed coefficient, and K_T_ is the temperature-sensed coefficient. The FBG-based temperature sensor was sealed with a protection cover to avoid stress affection and therefore Δε can be set to zero.

In Equation (1), where changes in variables of n_eff_ and Λ are independent defined as Δn_eff_ and ΔΛ that determines the variation in λ_B_, as shown in Equation (3):

(3) $$\Delta \lambda_{B}=2\Lambda \Delta n_{eff}+2n_{eff}\Delta \Lambda$$

The optic fibers were polished and etched to remove fiber cladding; hence, the bare fiber core can contact the ambient environment condition directly. This increases the changes in the n_eff_ value, resulting in a noticeable wavelength shift. Moreover, the polishing process does not change the period of the grating; hence, ΔΛ can be set to zero.

In the NH_3_ sensor, the sensitive thin film was coated on the etched flat top of FBG. The thin film absorbs external substances and changes the refractive index or grating period, thus shifting the reflection center wavelength and increasing the sensitivity of the sensor. Generally, nano sols are used as the thin film coating because it is a hydrophilic compound that can expand by absorbing water or contract by releasing water. Such an expansion or contraction process is converted to a change in force or pressure that induced sensible responses.

### 2.2. Long-Period Fiber Grating

The LPG is also called the transmission fiber grating, a period of which ranges from tens to hundreds of micrometers. If the wave vector of the refractive index period modulation has fulfilled the phase matched condition, then light from the fundamental fiber core mode, was transmitted along the optical fiber axis to couple into multiple cladding modes, thus would create numerous loss peaks and resonant peaks on the transmission spectrum. The modulation with a large period will enable coupling light from a fiber core into its cladding. Since such a modal change dissipates most of the light energy, only the wavelength of light having an identical traveling direction, but dissimilar mode, can propagate forward, resulting in a filtering effect.

A light filter is a wavelength-selected component. When a light wave segment enters a fixed-wavelength filter, only wave signals with the target wavelengths can pass through, and the remaining signals are excluded. In this study, a FBG was designed as the sensing end of the proposed optical fiber sensing array. The FBG was linked to the LPG to form a mixed-type fiber grating. The reflection center wavelength of the FBG was arranged to the positive or negative slope area that was induced by the loss peak of the LPG. The fiber grating-linked device was placed in a designed package framework, thus achieving simultaneous measurement and filtering.

### 2.3. Fluorescent Reaction

An optical fiber was coated with a sensitive nano sol to form an optical fiber fluorescent sensing component. The input light can be reflected on the interface between a fiber core with a high refraction index and a nano sol with a low refraction index. An evanescent field is derived through total reflection, exciting the sensitive fluorescent material to create a measurable fluorescent signal [44]. Generally, the excitation wavelength is determined by the energy gap of the material. The energy excited by the input light source is usually higher than the emitted fluorescence, and the wavelength of the light source is shorter than the luminescence-emitted wavelength. A follow up filter is used to exclude noise from the light source. Since fluorescent light is affected by a specific gas to induce a quenching reaction to decrease the intensity of the luminescence, a frequency matched light detecting device is applied to measure luminescence intensity from designed sensing array. This device converts a luminescence signal to an electrical analog signal, facilitating subsequent measurement and integration.

## 3. Fabrication of the Gas Sensing Array

**Figure 1:**
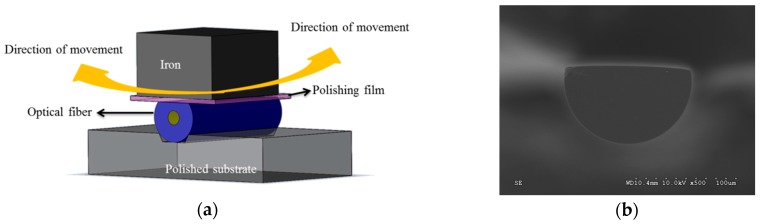
(**a**) Framework for polishing the optical fibers; and (**b**) cross-sectional image of a polished fiber.

The proposed sensing array featured two types of sensor, one of which was used to measure O_2_ based on fluorescent quenching and CO_2_ based on light intensity variation. The other applied a fiber grating connected to a filter to measure ambient temperature and NH_3_ concentration. To increase the sensitivity of the sensing array, the sensing areas of the fibers were etched and polished before coating with the nano sol film, thus decreasing the interference from external cladding substances. Since optical fibers can endure a large axial tension but break easily under a lateral stress, the optical fibers used in this study were polished along an axial direction. The polishing depth of the fiber was 41.87 ± 0.25 μm. The polished experiments were controlled in polishing time, grind particle size, and optical power loss. Figure 1 illustrates the framework for polishing the optical fibers and a cross-sectional image of a polished fiber taken by an electron microscope.

### 3.1. Sol-Gel Method

When organic or inorganic compounds are hydrolyzed, condensed, gelatinized, and heat-dried, the resulting products exhibit advantages including high purity, low synthesis temperature, stable chemical property, and controllable composition [45]. Rapidly hydrolyzing an inorganic salt or silicon alkoxide forms a sol with densely packed particles, the surface charge repulsion of which is removed through solvent evaporation. Next, the sol pectizes and then changed into a colloidal gel. Based on developed results, the reactions of hydrolysis and condensation can be described. The hydrolysis reaction is shown in Equation (4):

Si(OR)_4_ + H_2_O → HO-Si(OR)_3_ + ROH (4)

where R is an alkyl group such as CH_3_, C_2_H_5_, or C_3_H_7_, and ROH is an alcohol.

The hydrolysis reaction is accompanied by condensation reaction, resulting in molecules bonding together. The reaction proceeds continuously in the system, forming expanding molecules with silicon atoms. The condensation reaction equation is expressed as Equation (5):

(OR)_3_Si-OH + HO-Si(OR)_3_ → (OR)_3_Si-O-Si(OR)_3_ + H_2_O (5)

The condensation product covers the entire reaction solution and forms a sol. The sol-gel method generally involves dissolving an organic metal or a metallic alcohol in an organic solvent to form an initiating solution. When the hydrolysis reaction starts, the condensation reaction occurs spontaneously, forming a four-sided SiO_2_ web-shaped structure. Byproducts of alcohol and water must be removed afterward.

### 3.2. Nano Film Preparation

TEOS was used as the precursor for forming supporting structure of the thin film. This compound material reacts with water easily to undergo hydrolysis and condensation. Ethanol was used as a solvent to resolve the hydrophobicity of the alkyl compound and the reaction occurred in a homogenous phase. The variation of pH value affects the hydrolysis and condensation of TEOS as well as the size of the resulting particles. Consequently, HCl was used as a catalyst to adjust pH value during thin film process. The solution was prepared based on the reaction of TEOS, ethanol, deionized water, and HCl at room temperature. Final optimal parameters for synthesizing the nano sol film were determined based on the following conditions: HCl concentration 1.96 × 10^−1^ mL, ethanol amount 10 mL, [H_2_O]/[TEOS] molar ratio of 0.5, and drying temperature of 20 °C.

### 3.3. CO_2_ and O_2_ Sensors

The CO_2_ and O_2_ sensors were developed based on optical fiber fluorescence reaction. The luminescence from sensitive fluorescent materials can be quenched by O_2_ which is applied to design O_2_ sensor. The CO_2_ concentration is measured based on the fluorescent intensity change from the pH indicator, which can convert the CO_2_-dependent absorbance to fluorescence intensity-based signal. The luminescence intensity was measured by a photodetector to produce optical signal which was converted into electrical signal by an optoelectrical coupling circuit for subsequent system-level measurement.

The nano sol thin film processes of CO_2_ and O_2_ sensors are described as following: (1) ethanol, deionized water, and HCl were mixed and stirred for 5 min. (2) TEOS was then added and stirred for 10 min. (3) A fluorescent material was added to the mixture and continuously stirred for 2 h until the material was completely dissolved to form a sensitive nano sol. The fluorescent material emits luminescence that is only inhibited by O_2_, which was composed of 0.2 × 10^−1^ mL Tris(2,2′-bipyridyl) dichlororuthenium(II) hexahydrate and Tris(bipyridine)ruthenium(II) chloride [46]. By contrast, 0.5 mg 1-hydroxy-3,6,8-pyrene trisulfonic acid trisodium salt was used as the fluorescent material to detect CO_2_.

To ensure sensing thin film was effectively excited by the evanescent field, the optical fiber was etched before coating a sensing thin film. The fiber cladding was etched using HF to expose the core. Following HNO_3_ was used to etch fiber for 10 min. This activated the hydroxyl groups on the fiber surface to ensure the nano film of sol can be deposited on the fiber easily. The final diameter of the isotropically etched fiber was 32 μm. Then, the optical fiber was dipped into the sol at constant speed of 3 mm/s controlled by a precision displacement platform. The nano sol solidifies on the etched top of the optical fiber to form a 1 cm sensing structure.

### 3.4. Temperature and NH_3_ Sensors

The FBG and LPG integrated structures were separately designed for sensing temperature and NH_3_. The FBG served as the sensing end, whereas the LPG served as the filter. The temperature or NH_3_ sensing structure was fabricated by connecting the LPG and FBG in series, where the FBG sensed signal is demodulated from the LPG.

**Figure 2:**
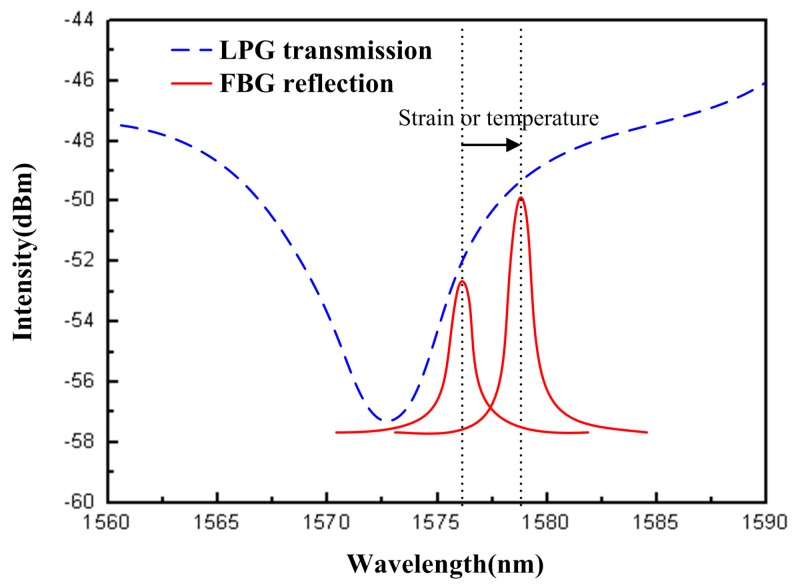
The schematic spectra of the LPG and FBG structures.

The sensing principle is based on varied light intensity measurement. An LPG is used to filtering the signal from the FBG sensing end. The schematic diagram of transmission spectrum of LPG and reflection spectrum of FBG is shown in Figure 2. The loss bands of LPG transmission spectrum create a linear useful spectrum region with a wide range. The reflection central wavelength of FBG is arranged on the loss bands of the LPG transmission spectrum. When the FBG sensing end is modulated by a variation of strain or temperature, the reflection central wavelength of FBG will shift. There is a relative shift between the reflection central wavelength of FBG and the loss bands of LPG transmission spectrum. The reflected light intensity from FBG was measured by a photodiode. The measured light power of the photodiode had altered linearly with the relative reflection central wavelength shift. Therefore, the reflection center wavelength of FBG sensing end has redshifted due to the variation of strain or temperature, hence, the measured light power of the photodiode increases. Otherwise the measured light power of the photodiode has reduced.

The FBGs were inscribed on hydrogen-loaded single-mode fibers (SMF-28, Corning, New York, NY, USA) using phase masks and a light source of a 248 nm KrF excimer laser (L1-FBG, Lambda Physik, Fort Lauderdale, FL, USA) and an amplified spontaneous emission (ASE) (ASE-FL7002, THORLABS, Newton, NJ, USA). An optical spectrum analyzer (OSA) (Q8384, ADVANTEST, Tokyo, Japan) was used to examine the characteristics of the inscribed gratings. The reflection center wavelengths of the FBGs were arranged at 1551.82 and 1554.16 nm. The amplitude masks were used to inscribe the LPGs on boron-doped photosensitive fibers (PS1250/1500, Fibercore, Southampton, UK). The transmission spectra of the completed LPGs exhibited multiple loss peaks. The center wavelengths of the most intense transmission sites were 1550.46 and 1554.63 nm.

The optical fiber of the NH_3_ sensor underwent a one-sided polishing process as follows: first, the inscribed fiber grating was placed and fixated in a groove of polymethylmethacrylate (PMMA) substrate. Next, the grating was polished using a rough diamond lapping film (UND 3) followed by a fine diamond lapping film (UND 0.5). Finally, the PMMA substrate was placed in acetone to dissolve the epoxide from the fiber. Alcohol was used to clean the polished side of the fiber.

To check the pH value of samples, 3 indicators were added to extend measurable pH range that did not influence NH_3_ measurement results [12,47]. The NH_3_ sensing film was prepared by adding 0.4 mg chlorophenol red, 0.5 mg bromophenol blue, and 0.4 mg cresol red into 72.5 g nano sol. The mixture was stirred for 1 h, and then adjusted its pH value to neutral using HCl [47,48,49]. The sensing film was coated using a precision displacement instrument. Different concentrations of NH_3_ can change reaction pH values of the dyed thin film to excite dissimilar wavelengths; hence, the NH_3_ concentration can be measured by determining the wavelength variations from the optical reaction.

### 3.5. Design of Measurement System

Two light sources were applied to excite the proposed sensing array. A light source with a visible wavelength 470 nm (M470L3, THORLABS, Newton, NJ, USA) is used to excite the fluorescence of O_2_ (610 nm) and CO_2_ (520 nm) sensing thin films. Another 1500 nm amplified spontaneous emission (ASE-C-10S, FiberLabs, Saitama, Japan) is used for the NH_3_ and temperature sensors.

**Figure 3:**
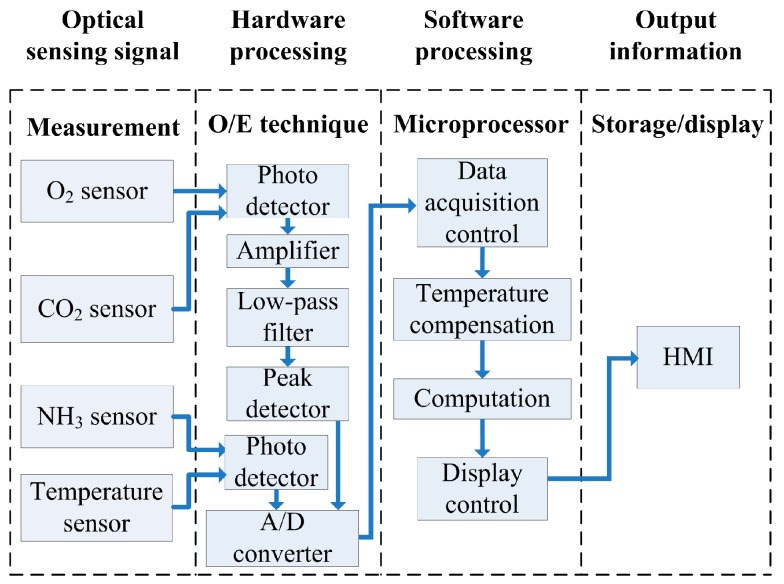
Block diagram of the optical fiber gas sensing array measurement system.

**Figure 4:**
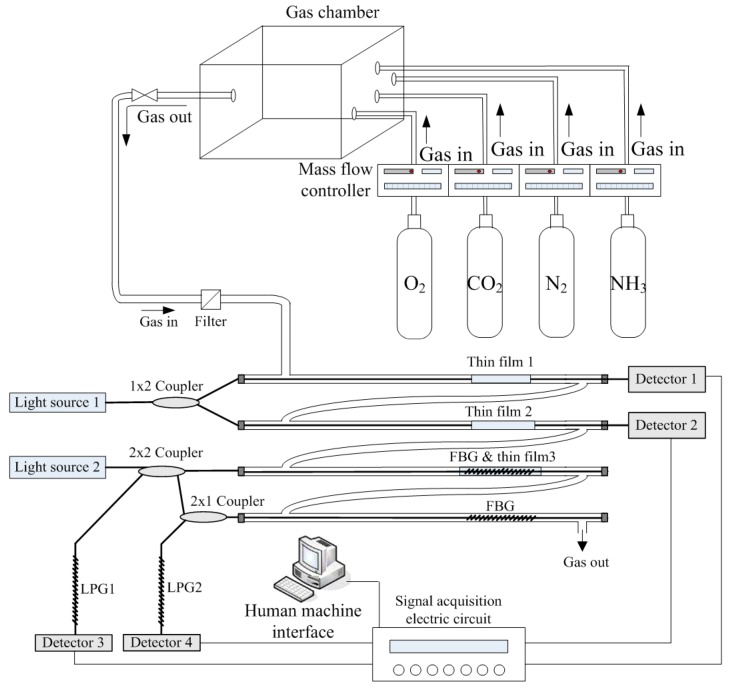
Optical fiber sensing array gas measurement system.

The optical signals detected by the sensing array were processed by an optocoupler-and-convertor circuit for transmitting analog voltage signals to a microprocessor (Arduino UNO R3, Smart Projects, Milano, Italy). The optocoupler circuit was designed to convert luminescence into an electric signal as follows: first, the internal resistance of the photodiode was selected to match with the sensing resistance for catching better induced incident luminescence photocurrent which will be converted into voltage signal and transmitted into a buffer circuit. The transformed voltage was very weak, which must be amplified suitably. Therefore, an amplifier with a function of adjustable gains was designed to amplify the weak signals. A second-order low-pass filter was designed to eliminate the high frequency noise. The filtered sinusoidal voltage signal was transmitted into a peak-and-hold circuit to obtain a stable DC voltage. Finally, the maximum signal obtained from the peak-and-hold circuit was transmitted into a buffer amplifier for following analog-to-digital conversion. A microprocessing circuit then converted the processed voltage signals into 10-bit digital data to conduct calculation and analysis. Finally, the processed data were transmitted to a human-machine interface (HMI) through Bluetooth communication and then displayed as graphical outputs. The main program features three analyzing formulas, namely initial adjustment, temperature compensation, and measurement termination. Before a sample was measured, an external interruption mechanism was initiated to detect ambient temperature for calibrating temperature compensation. A continuous sampling technique was applied with one second of sampling time for measuring concentrations of different gases simultaneously. The stored data in text file can be displayed by word and diagram types. Figure 3 illustrates the overall framework of the measurement system. Figure 4 illustrates the measurement configuration of the system used to detect O_2_, CO_2_, and NH_3_ concentrations simultaneously in real-time.

## 4. Measurement Analysis and Discussion

To excite the proposed optical fiber gas sensing array, two sets of light sources coordinated with three optocouplers were arranged. The light sources were divided and transmitted to four optical fiber sensors that generated different operation light waveforms. Moreover, the sensing films were composed of distinctive fluorescent materials and dyes, resulting in distinctive fluorescent emission segments and wavelengths. Therefore, the proposed sensing array can simultaneously measure concentrations of O_2_, CO_2_, NH_3_, and temperature.

### 4.1. Analysis on Nano Sensing Structure

**Figure 5:**
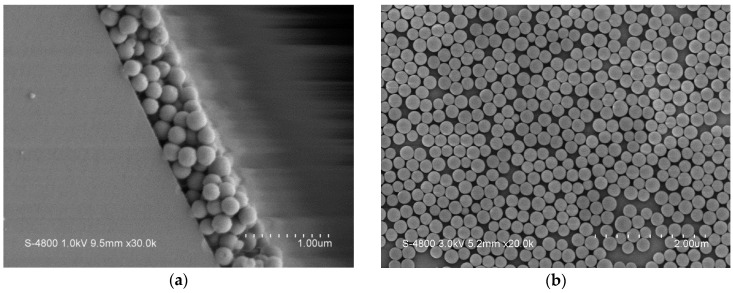
(**a**) A SEM image of the nano particles of the sensing thin film on the optical fiber surface; (**b**) the nano particle size and coverage are 200 nm and 73%.

The nano sol was mainly synthesized through hydrolysis and condensation reactions. Specifically, TEOS mixed with deionized water were hydrolyzed and condensed to form the sol containing nano silica particles. Additionally, conditions including environmental temperature, catalysts, and solvents also affect the properties of the nano sol. The nano sol films were fabricated based on experimental parameters that were analyzed using scanning electron microscope (SEM) as shown in Figure 5. According to results, the average size of the silica particles was 200 nm, moreover, the nanoparticle-covered area was about 73% of the sensing area. Both thicknesses of the nano sol films for sensing O_2_ and CO_2_ were all the same about 625 nm.

### 4.2. Gas Sensing Measurement and Analysis

**Figure 6:**
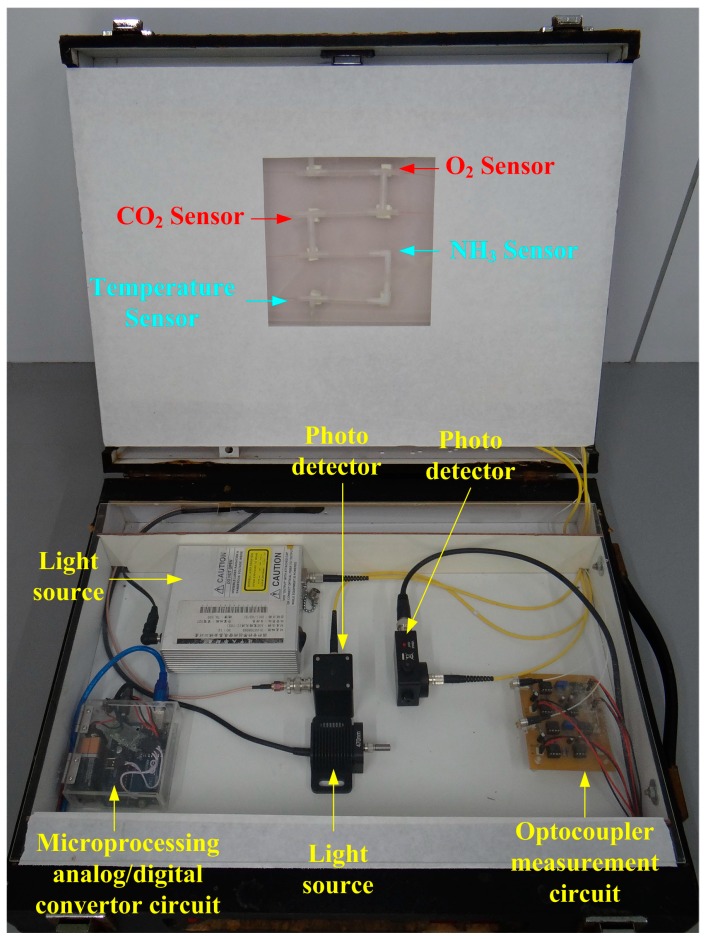
The photo of a prototype portable instrument.

A measurement system of the proposed sensing array was composed of the light sources, optical fiber sensing components, signal processing circuits, and optical detectors. The arranged optocouplers have separated the sensing array signals to measure different gases simultaneously. The experimental setup consisted of a gas sensor array, optical convertors, and optocoupler measurement circuits, as shown in Figure 6. Pure nitrogen gas was used as the background and purge gas. Mass flow controllers were adopted to mix gases for controlling gas concentrations. The experiments were conducted under normal pressure and room temperature. The gas samples were evenly mixed in a gas chamber to have a stable test concentration then injected into the sensing array to conduct measurement. Since the sensing mechanism relies on pH variation, the NH_3_-induced wavelength changes could be also produced by NO_2_. The cross-sensitivity of the device to different indicator did not addressed in this work. Our experiments show that O_2_ and CO_2_ sensing cannot be influenced by NH_3_ or other atmosphere gases.

**Figure 7:**
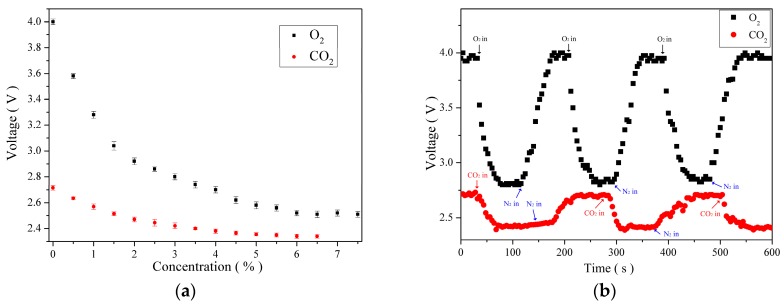
Analysis on the O_2_ and CO_2_ sensors: (**a**) voltage response curve relative to gas concentration; and (**b**) dynamic response time and recovery time measurement.

Figure 7a illustrates the measurement response curves of the O_2_ and CO_2_ sensors. The results revealed that when O_2_ or CO_2_ concentration increased, the excited fluorescent intensity decreased. The O_2_ response exhibited a quadratic curve behavior which was described piecewise using linear approximation. Specifically, the O_2_ sensor exhibited favorable sensitivity at a low concentration, but it decreased as the gas concentration increased. When the O_2_ concentration reached 6%, the sensed voltage response nearly saturated, indicating that the sensing range of the O_2_ sensor was less than, or equal to, 6%. Applying a two-segment analysis on the O_2_ sensor revealed that its sensitivity was 1.54%/V when the concentration was less than 1.5%, and it was 9.62%/V when the concentration ranged between 1.5% and 6%. The measurement response of the CO_2_ sensor was similar to that of the O_2_ sensor, the response slope gradually decreased as the gas concentration increased. When the CO_2_ concentration reached 5%, the sensing response nearly saturated, indicating that the sensing range of the CO_2_ sensor was less than 5%. When CO_2_ concentrations were less than 2% or between 2% and 5%, the sensitivity of the CO_2_ sensor was 8.33%/V or 27.78%/V, respectively. Figure 7b displays the response time and recovery time of the O_2_ and CO_2_ sensors. A testing gas composed of pure NH_3_ and 3% O_2_ (or CO_2_) was used to measure the dynamic time response. The results revealed that the response and recovery times of the O_2_ sensor were 120 ± 6.1 and 195 ± 8.9 s, respectively, and those of the CO_2_ sensor were 48 ± 2.6 and 76 ± 4.6 s, respectively. These measurements were conducted through gas diffusion to ensure even diffusion of gas samples. Therefore, the response time measured in this study incorporated gas diffusion time.

**Figure 8:**
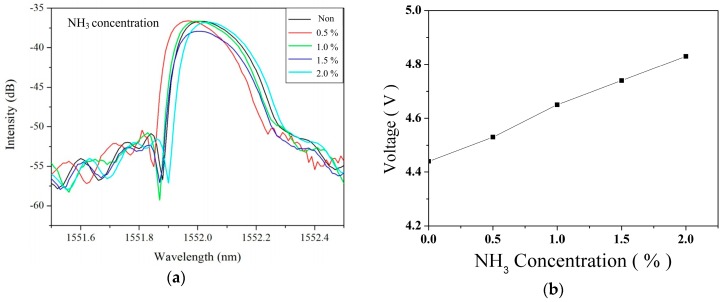
(**a**) The red shifts of the FBG sensing spectra induced by various NH_3_ concentrations; and (**b**) the sensed relationship between NH_3_ concentration and voltage response.

**Figure 9:**
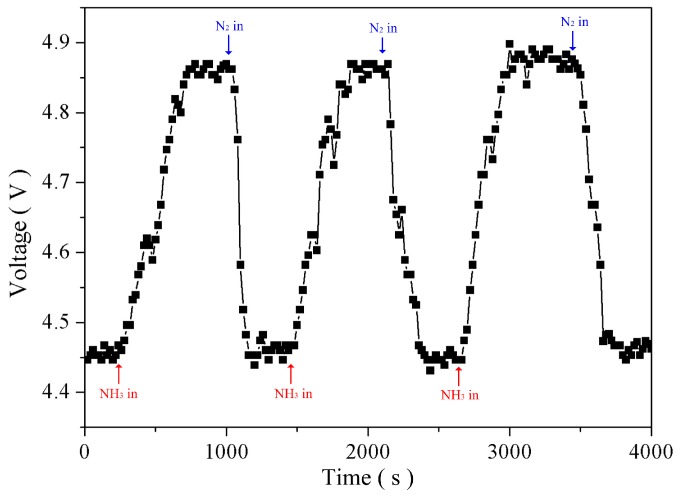
Dynamic characteristic of the NH_3_ sensor for analyzing the response time and recovery property.

Various concentrations of NH_3_ samples were prepared and inspected under room temperature that had shown the red shift phenomenon of the FBG reflection center. Each experimental repetition involved increasing the NH_3_ concentration by 0.5% in Figure 8a. When the NH_3_ concentrations were 0.5%, 1.0%, 1.5%, and 2.0%, the red shift values were 1551.970, 1552.000, 1552.010, and 1552.030 nm, respectively, measured using an OSA. The result is shown in Figure 8b that the sensitivity of the NH_3_ sensor is 0.2%/V. Figure 9 presents 390 ± 19.8 s in response time and 260 ± 15.6 s in recovery time for NH_3_ sensing measurement.

**Figure 10:**
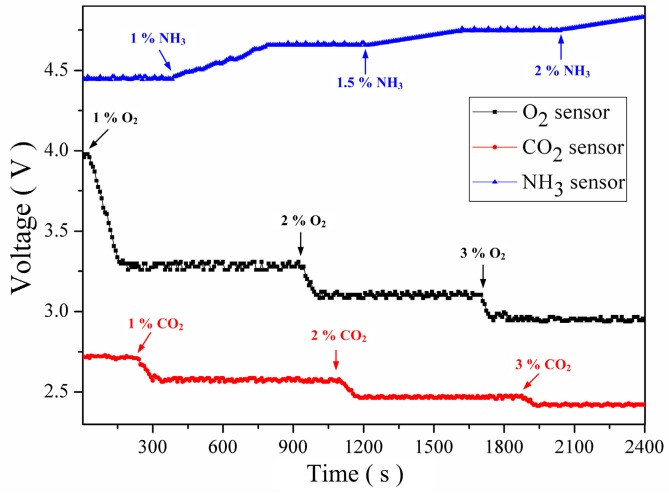
Simultaneous measurement of the voltage responses of the O_2_, CO_2_, and NH_3_ sensors.

Finally, an experiment was conducted to simultaneously measure concentrations of O_2_, CO_2_, and NH_3_ and examine selectivity of the gas sensing array. The measurement system linked the sensing array was cleaned using pure nitrogen gas under room temperature before and after each test cycle. Mixed concentrations of 1%, 2%, and 3% O_2_, CO_2_, and NH_3_ were sequentially injected into measurement chamber, and the resulting signal variations were processed by the measurement system. The result is shown in Figure 10. When O_2_ was injected, the response voltage of the O_2_ sensor noticeably decreased, whereas those of the CO_2_ and NH_3_ sensors remained unchanged. When CO_2_ was injected, the response voltage of the CO_2_ sensor markedly decreased, whereas those of the O_2_ and NH_3_ sensors remained unchanged. When NH_3_ was injected, the response voltage of the NH_3_ sensor substantially increased, whereas those of the O_2_ and CO_2_ sensors remained unchanged. The results indicated that the sensors in the sensing array keep proper single gas sensing function unaffected by other gases, indicating favorable gas selectivity.

### 4.3. Temperature Compensation

Ambient temperature influences gas measurement responses of the sensing array, thus changing the output voltage values, which are mixed and converted from the sensing signals. Consequently, temperature compensation must be applied to correct the output values of the sensors. The temperature compensation tests were conducted under 20 °C, 40 °C, and 60 °C to examine temperature effect of O_2_, CO_2_, and NH_3_ concentration measurements and acquire compensation parameters. The concentrations of gas samples were arranged between 0% and 3%. A linear function shown as Equation (6) was used to determine the relationship between temperature variation and voltage response:

V(T_x_) = V(T_0_) + C_X_(T_0_ − T_x_) (6)

where V(T_0_) is the voltage response at 20 °C; V(T_x_) is the voltage response at the ambient temperature; and C_X_ is the temperature compensation parameter. The results revealed that the temperature compensation parameters of the gases of O_2_, CO_2_, and NH_3_ at a low concentration were −7.25 mV/°C, −9.0 mV/°C, and 10.5 mV/°C. These parameters were used to ensure concentration measurements unaffected by temperature.

**Figure 11:**
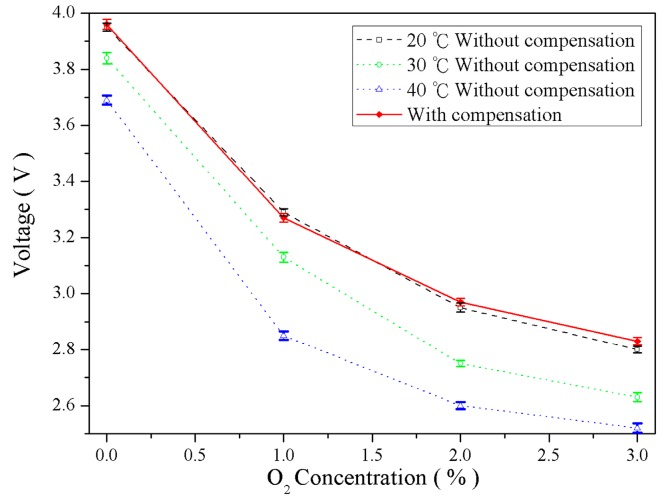
Comparison of O_2_ concentration–voltage response relationships before and after temperature compensation.

**Figure 12:**
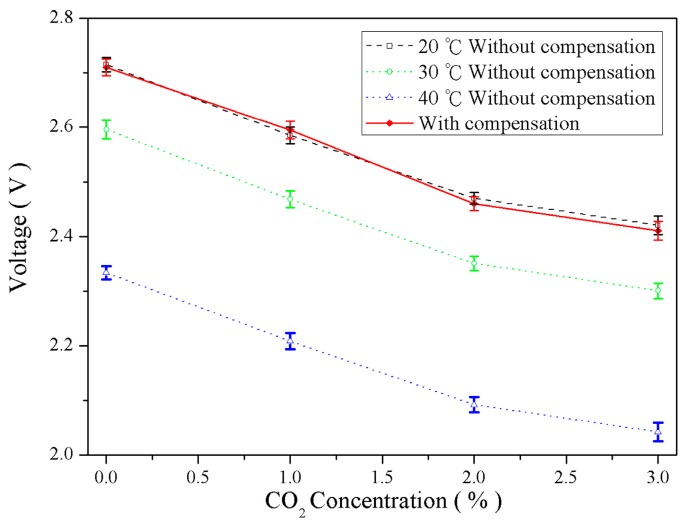
Comparison of CO_2_ concentration–voltage response relationships before and after temperature compensation.

**Figure 13:**
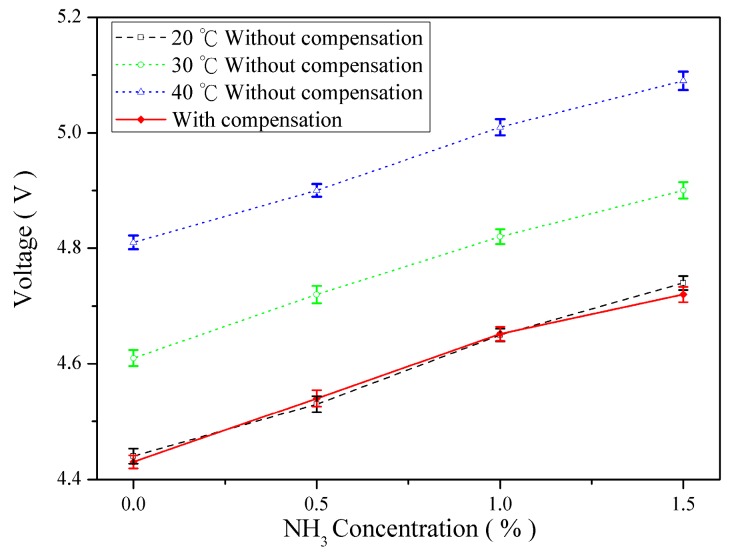
Comparison of NH_3_ concentration–voltage response relationships before and after temperature compensation.

Figure 11, Figure 12 and Figure 13 illustrate the gas concentration-and–voltage relationships for O_2_, CO_2_, and NH_3_ sensors before and after temperature compensation. The results show that without temperature compensation the temperature dependent separations in voltage responses are large. The temperature-induced deviation in sensing O_2_, CO_2_, and NH_3_ were averaged 11.3%, 18.1%, and 8.6%, respectively. After temperature compensation, the gas voltage responses were not influence due to temperatures fluctuation, and the averaged error of O_2_, CO_2_, and NH_3_ were reduced to 2.9%, 1.24%, and 0.47%, respectively. The accuracy of O_2_, CO_2_, and NH_3_ was improved 8.4%, 16.9%, and 8.13%, respectively.

The results revealed that the gas concentration and temperature measured by the proposed sensing array were linearly and inversely correlated. The proposed system can be used to measure gas concentrations under various temperatures to obtain accurate gas concentrations on the designed human machine interface. Moreover, a temperature compensation technique was applied to drastically decrease errors incurred by temperature fluctuation, thus further increasing the accuracy of the sensing array.

## 5. Conclusions

This study developed a portable gas measurement instrument with temperature compensation based on an optical fiber sensing array. Through examining the relationship of reflection wavelength shift with gas concentration and fluorescent intensity, the concentration responses of O_2_, CO_2_, and NH_3_ can be accurately measured by the proposed sensing array. This array is also incorporated with a temperature sensor that provides temperature compensation for calibrating gas concentrations. The averaged diameter of sensing sol-gel particles was 200 nm and the surface coverage ratio was 78%. The sol synthesis parameters were 0.5 molar ratio of H_2_O to TEOS, and 1.96×10^−1^ mL of HCl at 20 °C. The sampling peak-and-hold circuit measures the related maximum value of gas responses without affection of operation deviation, thus, effectively decreasing measurement errors. After a linear approximation, the sensitivity of the oxygen sensors were 1.54%/V and 9.62%/V for concentrations less than 1.5% and for concentrations between 1.5 and 6%, respectively. The sensitivity of the carbon dioxide sensors were 8.33%/V and 9.62%/V for concentrations less than 2% and for concentrations between 2 and 5%, respectively. For the ammonia sensor, the sensitivity was 27.78%/V, while ammonia concentration was less than 2%. The response and recovery times were, respectively, 120 ± 6.1 and 195 ± 8.9 s for the O_2_ sensor; 48 ± 2.6 and 76 ± 4.6 s for the CO_2_ sensor; and 390 ± 19.8 and 260 ± 15.6 s for the NH_3_ sensor. The response time delays were partially caused by gas diffusion time, which can be decreased by designing a volume-reduced testing chamber with controllable local heaters. The proposed sensing array could possibly be used in incinerators and dump pits for detecting O_2_, CO_2_, and NH_3_ concentrations in real-time.
